# Hospitalisation and mortality in patients with comorbid COPD and heart failure: a systematic review and meta-analysis

**DOI:** 10.1186/s12931-020-1312-7

**Published:** 2020-02-14

**Authors:** Eleanor L. Axson, Kishan Ragutheeswaran, Varun Sundaram, Chloe I. Bloom, Alex Bottle, Martin R. Cowie, Jennifer K. Quint

**Affiliations:** 1 National Heart and Lung Institute, Imperial College London, G05 Emmanuel Kaye Building, Manresa Road, London, SW3 6LR UK; 2 Dr Foster Unit, Department of Primary Care and Public Health, Imperial College London, London, UK

**Keywords:** COPD, Heart failure, Mortality, Hospitalisation, Rehospitalisation, Systematic review, Comorbidity

## Abstract

**Background:**

Discrepancy exists amongst studies investigating the effect of comorbid heart failure (HF) on the morbidity and mortality of chronic obstructive pulmonary disease (COPD) patients.

**Methods:**

MEDLINE and Embase were searched using a pre-specified search strategy for studies comparing hospitalisation, rehospitalisation, and mortality of COPD patients with and without HF. Studies must have reported crude and/or adjusted rate ratios, risk ratios, odds ratios (OR), or hazard ratios (HR).

**Results:**

Twenty-eight publications, reporting 55 effect estimates, were identified that compared COPD patients with HF with those without HF. One study reported on all-cause hospitalisation (1 rate ratio). Two studies reported on COPD-related hospitalisation (1 rate ratio, 2 OR). One study reported on COPD- or cardiovascular-related hospitalisation (4 HR). One study reported on 90-day all-cause rehospitalisation (1 risk ratio). One study reported on 3-year all-cause rehospitalisation (2 HR). Four studies reported on 30-day COPD-related rehospitalisation (1 risk ratio; 5 OR). Two studies reported on 1-year COPD-related rehospitalisation (1 risk ratio; 1 HR). One study reported on 3-year COPD-related rehospitalisation (2 HR). Eighteen studies reported on all-cause mortality (1 risk ratio; 4 OR; 24 HR). Five studies reported on all-cause inpatient mortality (1 risk ratio; 4 OR). Meta-analyses of hospitalisation and rehospitalisation were not possible due to insufficient data for all individual effect measures. Meta-analysis of studies requiring spirometry for the diagnosis of COPD found that risk of all-cause mortality was 1.61 (pooled HR; 95%CI: 1.38, 1.83) higher in patients with HF than in those without HF.

**Conclusions:**

In this systematic review, we investigated the effect of HF comorbidity on hospitalisation and mortality of COPD patients. There is substantial evidence that HF comorbidity increases COPD-related rehospitalisation and all-cause mortality of COPD patients. The effect of HF comorbidity may differ depending on COPD phenotype, HF type, or HF severity and should be the topic of future research.

## Introduction

Patients with chronic obstructive pulmonary disease (COPD) are at greater risk of developing comorbid heart failure (HF) than the general population [1]. COPD and HF share common risk factors (e.g. smoking) and symptoms (e.g. dyspnoea), making it difficult to diagnose one disease in the presence of the other and to describe their interaction [2]. Previous studies have found large amounts of unrecognised HF in COPD patients [3]. When recognised, HF is diagnosed later in COPD patients than in patients without COPD [4] and cardiovascular medications are consistently under-prescribed in the COPD population [5]. COPD patients often experience acute worsening of symptoms requiring additional management, termed acute exacerbations of COPD (AECOPD), where severe exacerbations resulting in hospitalisation (COPD-related hospitalisation) or death [6, 7]. The effect of HF comorbidity on hospitalisation and death of COPD patients is not well understood.

Several studies have shown that COPD patients with HF experience more AECOPD and hospitalisations than those without HF [8–10]. Meanwhile, Jones et al. did not find an association between cardiovascular comorbidity and AECOPD or mortality [11]; however, a composite cardiovascular exposure may not be indicative of the effects of individual cardiac conditions on COPD patients. Patel et al. found that COPD patients with ischaemic heart disease, a precursor to HF, experienced longer exacerbations than COPD patients without ischaemic heart disease, but not more exacerbations [12]. Boudestein et al. found that COPD patients with newly diagnosed HF experienced greater mortality [13]; however, a smaller study by Plachi et al. found no difference in mortality between COPD patients with and without HF [14].

A systematic review was conducted by Müllerova et al. investigating the association between various cardiovascular diseases, including HF, and outcomes for COPD patients compared with controls without COPD [15]. COPD patients were found to be at greater risk for cardiovascular-related hospitalisation compared with non-COPD controls [15]. Associations between COPD status and ischemic heart disease-related hospitalisation were not statistically significant, while the associations between COPD status and arrhythmia-related or stroke-related hospitalisation were mixed [15]. Heart failure-related hospitalisations were significantly increased in COPD patients compared with non-COPD controls [15].

Knowing the effects of HF comorbidity on morbidity and mortality of COPD patients is important to inform the development of practice guidelines. Current COPD guidelines state that COPD patients with cardiovascular disease should be treated according to cardiovascular disease guidelines as if they did not have COPD [16–18]; however, this is not easily done. Shared symptomology of COPD and HF makes identification of HF in COPD patients complicated, and there is currently no guidance on the matter [19]. Unrecognized HF is unmanaged HF, but even the management of recognized HF in COPD patients is not informed by clinical trials, but rather is limited to small studies and is poorly disseminated [19]. COPD patients are consistently under-prescribed cardiovascular medications [5, 20].

We conducted a systematic review and meta-analysis investigating the effect of HF comorbidity on hospitalisation, rehospitalisation, and mortality in the COPD population.

## Methods

This protocol follows the Preferred Reporting Items for Systematic Reviews and Meta-Analyses Protocols (PRISMA-P) guidelines [21] and outlines strategies for study screening, data extraction, and analyses. This protocol was registered with the International Prospective Register of Systematic Reviews (PROSPERO) ([22]; registration number: CRD42018089534). The protocol for this systematic review was published in BMJ Open [23].

Briefly, MEDLINE and Embase were searched using a predefined search strategy [23] for randomized controlled trials and observational studies (cohort and case-control) assessing the impact of comorbid HF on hospitalisation, rehospitalisation, and mortality of COPD patients. Included studies needed to involve an exposed participant group with diagnoses of both COPD and HF. Additionally, studies must have included a comparator group of ‘unexposed’ individuals, those diagnosed with COPD but not HF. After searching, duplicate results were removed and the remaining articles were screened firstly by title and abstract and secondly by full text for fulfilment of inclusion criteria. Any studies rejected during the full text screening were recorded along with reason for rejection. Information regarding study population, exposure, comparators, outcomes, and design were extracted from all studies determined to meet the inclusion criteria using a pre-specified data extraction form.

The primary outcomes of interest were hospitalisation, rehospitalisation, and mortality of patients with comorbid COPD-HF as compared with patients with COPD alone. Hospitalisation and rehospitalisation encompassed both Accident and Emergency (A&E) attendance and admission to inpatient care. Studies must have reported risk ratios, rate ratios, odds ratios (OR), or hazard ratios (HR). Adjusted ORs are included here for description; however, they were not considered for inclusion in potential meta-analyses. This is because adjusted OR are not compatible with meta-analysis as their magnitudes cannot be compared between statistical models [24].

Risk of bias within individual studies was assessed using a method devised previously [25], looking at bias stemming from the selection of participants, the measurement of variables, and the control of confounding. Each component was assessed independently and rated from ‘moderate to high risk of bias’, ‘unclear risk of bias’, or ‘low risk of bias’.

Study selection, data extraction, and risk of bias assessment were undertaken by two authors independently (ELA & KR) with disagreements adjudicated by a third author (JKQ).

The I^2^ statistic was used to assess the level of heterogeneity and appropriateness for meta-analysis. Meta-analyses using inverse probability weighting were conducted to calculate a pooled effect estimate for hospitalisation, rehospitalisation, and mortality using the appropriate model (fixed or random effects based on level of heterogeneity). Funnel plots were used to assess the likelihood of reporting bias, and Egger’s test was used to test for asymmetry. If a meta-analysis proved inadvisable, due to an insufficient number of studies or high heterogeneity, a narrative synthesis of the data was conducted.

## Results

**Fig. 1:**
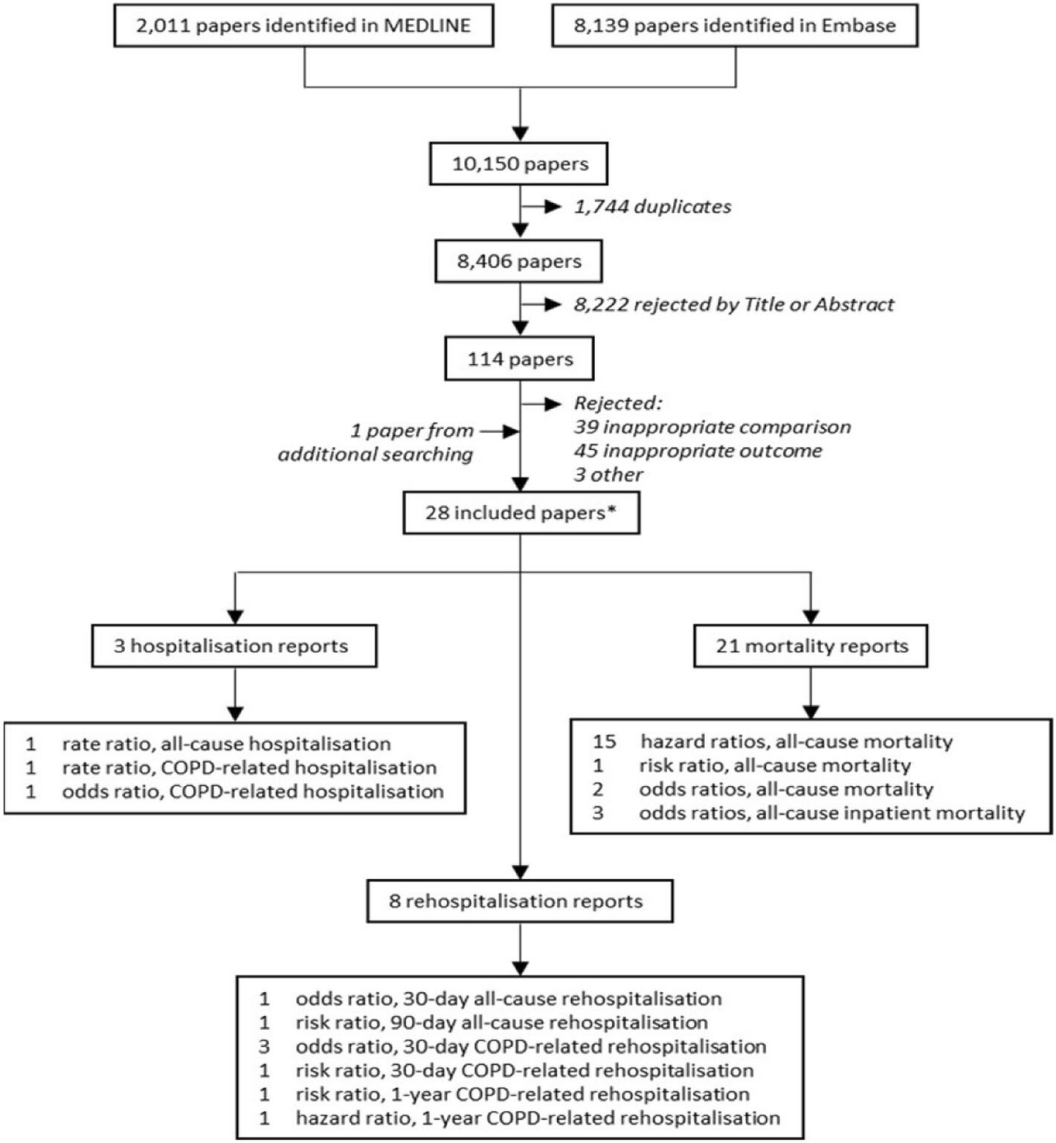
PRISMA flowchart for the study selection process. *Papers may have reported on mortality, hospitalisation, rehospitalisation, or any combination

**Fig. 2:**
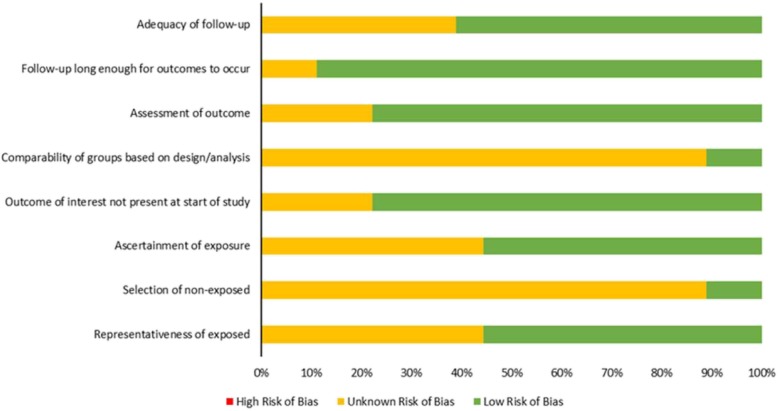
Risk of bias in individual studies

Following searching (Additional file 1: Figures S1 and S2) and screening, 28 articles were included in the review (Fig. 1). The majority of studies were from Europe and North America using electronic healthcare databases (Table 1, Additional file 1: Table S1). It was often difficult to assess the extent of bias due to poor reporting of methodology in many of the studies (Fig. 2).

**Table 1:** Description of included studies

Study Characteristics *N* = 28	
Year of Publication	
1980–1990	0
1991–2000	0
2001–2010	6
2011–2018	22
Geographic Region	
Africa	0
Asia	2
Australia/New Zealand	0
Europe	12
Multi-continent	2
North America	12
South America	0
Not Reported	0
Setting	
Single Centre	6
Multicentre (one country)	1
Multicentre (multi-country)	2
Database	19
Duration of Follow-Up	
< 1 year	5
≥ 1 and < 2 years	5
≥ 2 and < 3 years	3
≥ 3 years	9
Not Reported	6

### Identification of COPD

Eight studies explicitly stated requiring an forced expiratory volume in 1 s to forced vital capacity (FEV_1_/FVC) ratio of < 0.70 for a diagnosis of COPD [13, 26–32]. All studies were of adults; however, some studies set more specific age limitations for inclusion, the majority setting a minimum age [13, 26, 28, 30, 32–42]. Only three studies specified smoking history requirements for participants [26, 29, 30].

Five studies identified COPD patients through emergency visit or admission for COPD exacerbation as primary diagnosis [31, 41, 43–45]. For every emergency visit, clinicians may code multiple diagnoses, where the first listed diagnosis is considered the primary reason for the admission and each subsequent diagnosis consider a contributing factor to the admission. Genao et al. and Yeatts et al. required COPD coded in the first diagnostic position, or second position for Yeatts, for emergency visits [35, 36]. Perera et al. required COPD to be coded with concurrent diagnosis for pneumonia or mechanical ventilation [40]. Chen et al. required COPD to be coded in the top five diagnostic positions for emergency visit [34, 36]. Hasegawa et al. allowed COPD to be recorded in any diagnostic position [37]. Hoiseth et al. identified patients through dyspnoea as the primary complaint admitted through emergency, and an endpoint committee determined final diagnosis [46]. Carter et al. identified COPD patients from all patients admitted for any reason [47].

Many studies relied exclusively on electronic diagnostic coding for the identification of COPD or COPD-related hospital visits [33–42, 44, 45, 47–50]. Five studies allowed clinical diagnosis for the identification of COPD [13, 26, 30, 31, 46]. Two studies used a combination of electronic diagnostic coding and clinical diagnosis for COPD identification [43, 51].

### Identification of HF

Only two studies explicitly identified HF phenotype using classification by ejection fraction measures [13, 27]. Another two studies used medications as an indicator of HF diagnosis [29, 52]. Two studies relied on questionnaires only for identification of HF [28, 30], while Divo et al. used a combination of questionnaires, clinical diagnosis, and medication [29]. The majority of studies used electronic diagnostic coding [26, 31–45, 47–51] and/or clinical diagnosis [13, 29, 32, 43, 46, 51, 52] to identify HF.

### Description of the study populations

Twenty-one studies reported average age (standard deviation, SD) at start of follow-up, ranging from 56.6 ± 5.73 years [41] to 73.0 ± 5.3 years [13]. Chen et al. reported average age (SD) at start of follow-up for patients who went on to have a readmission as 73.5 ± 9.8 years and those who did not have a readmission as 71.6 ± 11.4 years [34]. Simmering et al. reported age groups by readmission status, with the majority of readmitted and non-readmitted patients aged 70–79 [42]. Genao et al. reported the median (interquartile range, IQR) age at start of follow-up as 77 (71, 83) years [35]. Yeatts et al. reported age in groups, with the largest age group at the start of follow-up being 65–69 year olds at 13.9% of the cohort [36]. Lau et al. reported that 70.2% of their derivation cohort was > 65 years old and 67.6% of their validation cohort was > 65 years old [39]. Age distribution was not reported by Löh et al. [50] or by Silver et al. [45].

All but one study, Bertens et al. [33], reported the sex distribution of their participants. The proportion of males in the included studies ranged from 41% [41] to 89% [28, 29] male. Lau et al. reported male-to-female ratios of 1.25:1 in their derivation cohort and of 1.24:1 in their validation cohort [39].

Four studies [26–28, 30] reported the proportion of current smokers in their cohorts, ranging from 36% [30] to 45.8% [27] current smokers. Three studies [26–28] reported the proportion of former smokers in their cohorts, ranging from 41.4% [27] to 94.2% [28] former smokers. Santibáñez et al. reported that 15.7% of their cohort were never smokers [32].

Two studies required a minimum number of pack-years of smoking history in order to be included. Abukhalaf et al. required a minimum of 20 pack-years smoking history [26] and Divo et al. required a minimum of 10 pack-years smoking history [29]. Four studies [26–28, 30] reported the average (SD) number of pack-years ranging from 43.1 ± 23.7 [26] to 55.5 ± 28 pack-years [28]. Boudestein et al. reported median (IQR) number of pack-years for COPD patients with HF as 25.0 (IQR: 1.6, 41.8) and for COPD patients without HF at 15.0 (IQR: 0.0, 38.1) [13].

Severity of airflow limitation varied greatly amongst studies. Baseline forced expiratory volume in 1 second (FEV_1_) predicted ranged from an average (SD) of 39 ± 17% [46] to 67 ± 28% [13] in the 10 studies that reported it [13, 26–31, 43, 46, 48, 52]. Boudestein et al. reported average (SD) baseline FEV_1_ predicted for COPD patients with HF as 81.7 ± 24.2% and for those without HF as 83.7 ± 25.9% [13]. Baseline FEV_1_ ranged from an average (SD) of 0.96 ± 0.43 L [46] to 1.19 ± 0.54 L [28] in the four studies that reported it [28, 31, 46, 52]. Eight studies reported on the proportion of patients with Global Initiative for Chronic Obstructive Lung Disease (GOLD) staging of airflow limitation severity [13, 26, 28, 29, 31, 32, 48, 51, 52], with a range of 11.2% [13] to 72.3% [52] of the study population being GOLD3–4. Boudestein et al. included 40% of the study population having GP-diagnosed COPD, but not meeting GOLD criteria [13].

### Hospitalisation

**Fig. 3:**
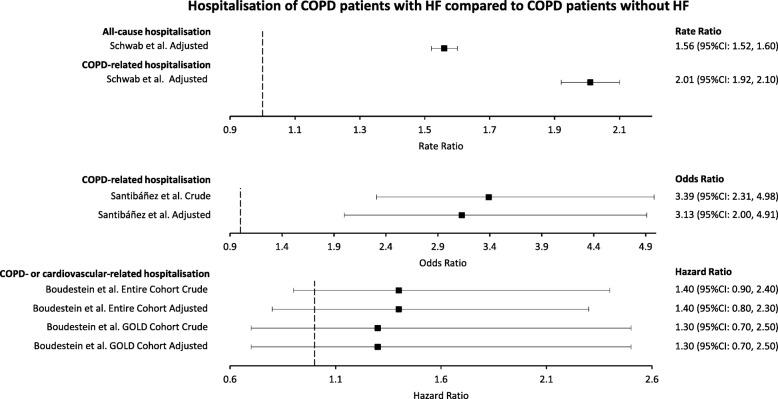
Estimates of the effect of HF comorbidity on all-cause and COPD-related hospitalisation of COPD patients. Heart failure (HF). Chronic obstructive pulmonary disease (COPD)

All studies reporting on hospitalisations compared COPD patients with HF with those without HF, where hospitalisation included attendance at A&E and/or admission to inpatient care (Fig. 3). Schwab et al. reported adjusted rate ratios for all-cause hospitalisation of 1.56 (95% confidence interval (CI): 1.52–1.60) and for COPD-related hospitalisation of 2.01 (95%CI: 1.92, 2.10) [49]. Santibáñez et al. reported a crude OR for COPD-related hospitalisation of 3.39 (95%CI: 2.31, 4.98), which slightly attenuated to 3.13 (95%CI: 2.00, 4.91) when adjusted [32]. Boudestein et al. reported a crude hazard ratio in their complete cohort for COPD- or cardiovascular-related hospitalisation of 1.40 (95%CI: 0.90, 2.40), which did not change following adjustment (HR = 1.40, 95%CI: 0.80, 2.30) [13]. Boudestein et al. reported a crude hazard ratio in their GOLD cohort for COPD- or cardiovascular-related hospitalisation of 1.30 (95%CI: 0.70, 2.50), which did not change following adjustment (HR = 1.30, 95%CI: 0.70, 2.50) [13]. Meta-analysis was not appropriate due to not enough studies reporting.

### Rehospitalisation

**Fig. 4:**
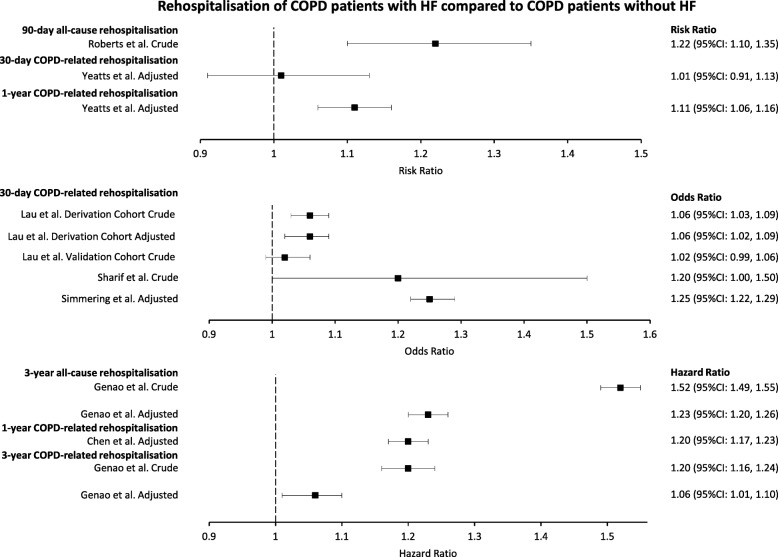
Estimates of the effect of HF comorbidity on all-cause and COPD-related rehospitalisation of COPD patients. Heart failure (HF). Chronic obstructive pulmonary disease (COPD)

All studies reporting on rehospitalisation required an index hospitalisation for COPD and compare COPD patients with HF with those without HF, where rehospitalisation included re-attendance at A&E and/or readmission to inpatient care (Fig. 4). Meta-analyses were not appropriate due to not enough studies reporting on a distinct outcome with the same measure.

Roberts et al. reported a crude risk ratio for 90-day all-cause rehospitalisation of 1.22 (95%CI: 1.10, 1.35) [43].

Yeatts et al. reported an adjusted risk ratio for 30-day COPD-related rehospitalisation of 1.01 (95%CI: 0.91, 1.13) [36]. Lau et al. reported a crude OR for 30-day COPD-related rehospitalisation of 1.06 (95%CI: 1.03, 1.09) in their derivation cohort and of 1.02 (95%CI: 0.99, 1.06) in their validation cohort [39]. In their derivation cohort, the adjusted OR for 30-day COPD-related rehospitalisation was 1.06 (95%CI: 1.02, 1.09). Sharif et al. reported a crude OR for 30-day COPD-related rehospitalisation of 1.2 (95%CI: 1.0, 1.5) [41]. Simmering et al. reported an adjusted OR for 30-day COPD-related rehospitalisation of 1.25 (95%CI: 1.22, 1.29) [42].

Chen et al. reported an adjusted HR for 1-year COPD-related rehospitalisation of 1.20 (95%CI: 1.17, 1.23) [34]. Yeatts et al. reported an adjusted risk ratio for 1-year COPD-related rehospitalisation of 1.11 (95%CI: 1.06, 1.16) [36].

Genao et al. reported a crude HR for 3-year all-cause rehospitalisation of 1.52 (95%CI: 1.49, 1.55), which attenuated to 1.23 (95%CI: 1.20, 1.26) after adjustments [35]. Genao et al. also reported a crude HF for 3-year COPD-related rehospitalisation of 1.20 (95%CI: 1.16, 1.24), which attenuated to 1.06 (95%CI: 1.01, 1.10) after adjustments [35].

### Mortality

**Fig. 5:**
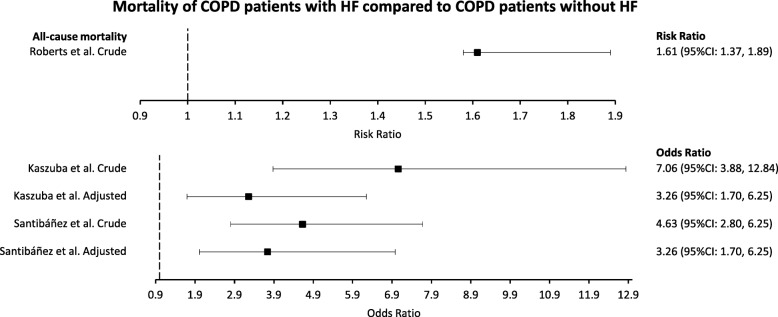
Risk and odds ratio estimates of the effect of HF comorbidity on all-cause mortality of COPD patients. Heart failure (HF). Chronic obstructive pulmonary disease (COPD)

One study reported risk ratio and one study reported odds ratio for all-cause mortality of COPD patients with HF compared with those without HF (Fig. 5). Roberts et al. reported a crude risk ratio of 1.61 (95%CI: 1.37, 1.89) for all-cause 90-day mortality comparing COPD patients with HF with those without HF [43]. Kaszuba et al. reported a crude OR of 7.06 (95%CI: 3.88, 12.84) for all-cause mortality, which attenuated to 3.26 (95%CI: 1.70, 6.25) when maximally adjusted [38]. Santibáñez et al. reported a crude OR of 4.63 (95%CI: 2.80, 7.68) for all-cause mortality, which attenuated to 3.74 (95%CI: 2.01, 6.98) when adjusted [32].

**Fig. 6:**
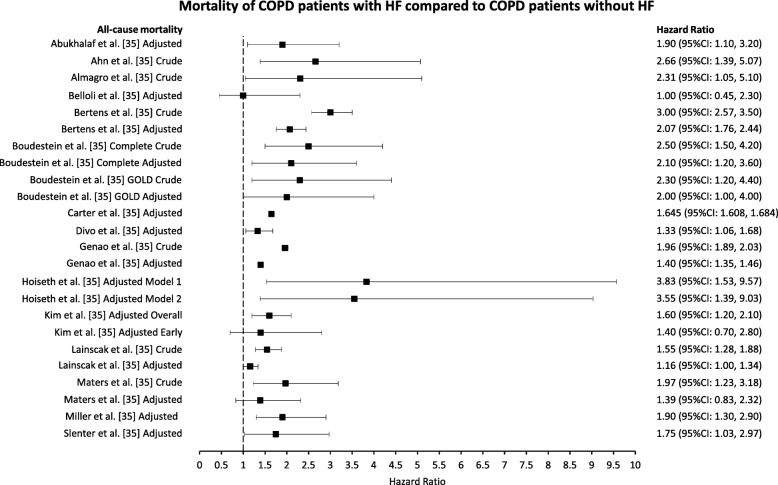
Hazard ratio estimates of the effect of HF comorbidity on all-cause mortality of COPD patients. Heart failure (HF). Chronic obstructive pulmonary disease (COPD)

**Fig. 7:**
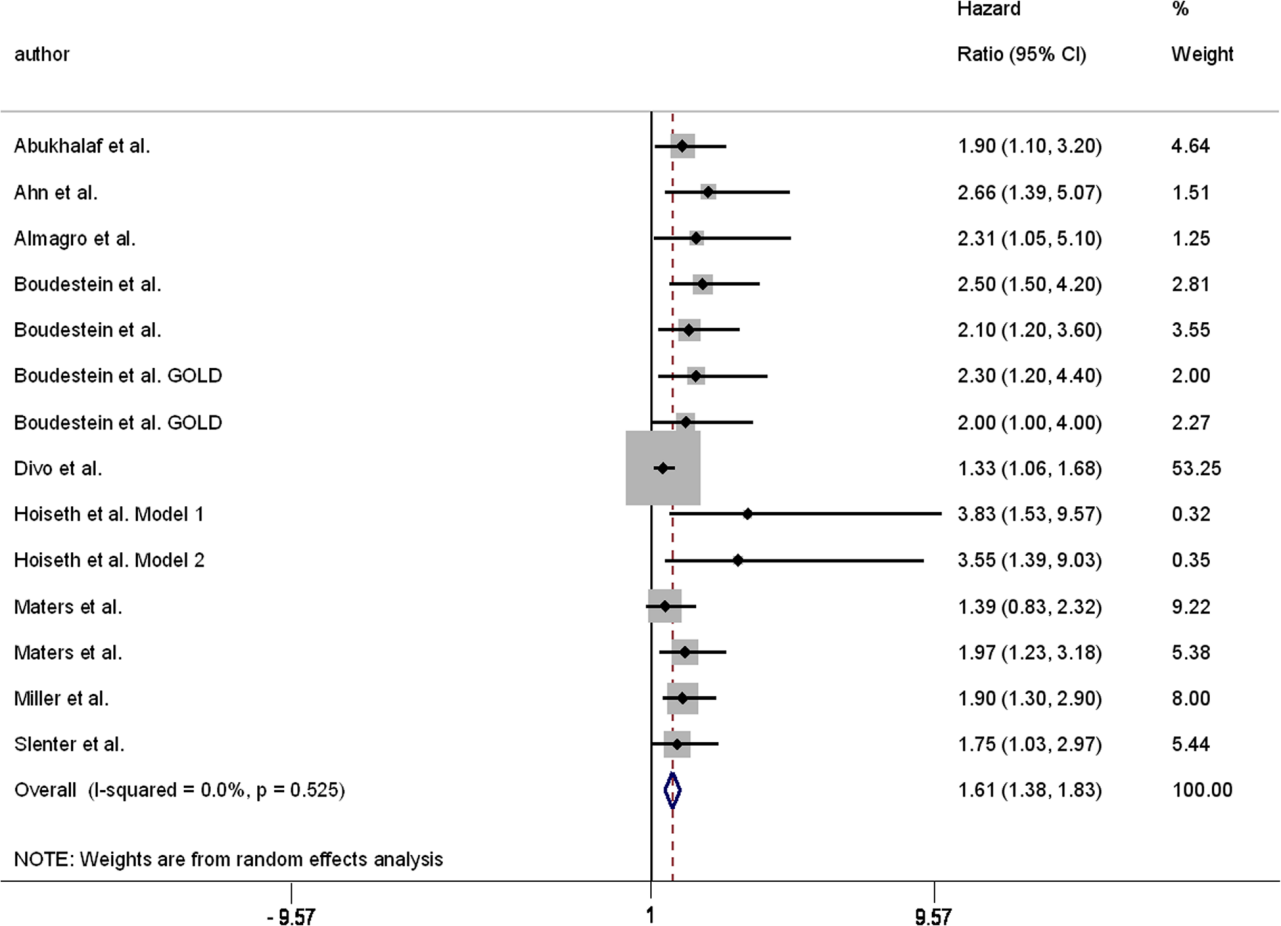
Pooled estimate for the effect of HF comorbidity on all-cause mortality of COPD patients diagnosed with spirometry. Heart failure (HF). Chronic obstructive pulmonary disease (COPD)

**Fig. 8:**
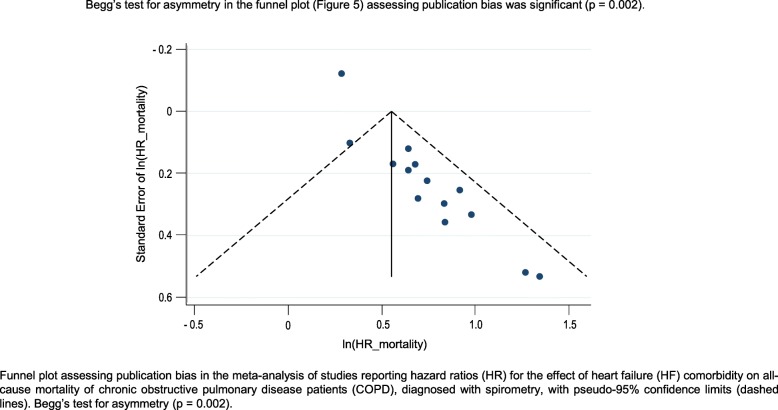
Funnel plot assessing publication bias. In the meta-analysis of studies reporting hazard ratios (HR) for the effect of heart failure (HF) comorbidity on all-cause mortality of chronic obstructive pulmonary disease patients (COPD), diagnosed with spirometry, with pseudo-95% confidence limits (dashed lines). Begg’s test for asymmetry (*p* = 0.002)

There were 15 studies reporting 24 HRs for all-cause mortality of COPD patients with comorbid HF compared with COPD patients without comorbid HF [13, 26–31, 33, 35, 44, 46–48, 51, 52] (Fig. 5). All crude HR estimates were statistically significant and ranged from 1.55 (95%CI: 1.28, 1.88) [51] to 3.00 (95%CI: 2.57, 3.50) [33]. Adjusted HR estimates ranged from 1.00 (95%CI: 0.45, 2.30) [48] to 3.83 (95%CI: 1.53, 9.57) [46], with five estimates failing to reach statistical significance [13, 44, 48, 51, 52]. Heterogeneity was considerable when considering all HR estimates (I^2^ = 90.3%). Stratifying by crude/adjusted status, length of follow-up, sample size, or whether patients were identified in primary or secondary care did not reduce heterogeneity (data not shown). When only studies that explicitly required an FEV_1_/FVC < 0.70 for a diagnosis of COPD to be made were analysed [13, 26–31, 46, 52], there was no heterogeneity (I^2^ = 0.0%). The pooled HR for the effect of HF on all-cause mortality of COPD patients, diagnosed using spirometry, was 1.61 (95%CI: 1.38, 1.83) (Fig. 6); however, there was evidence of publication bias in the funnel plot and from Begg’s test (*p* = 0.002) (Fig. 7). Heterogeneity was considerable when analysing studies that used electronic coding to identify COPD patients (I^2^ = 96.0%). A funnel plot showed evidence of publication bias (Fig. 8).

### Inpatient mortality

**Fig. 9:**
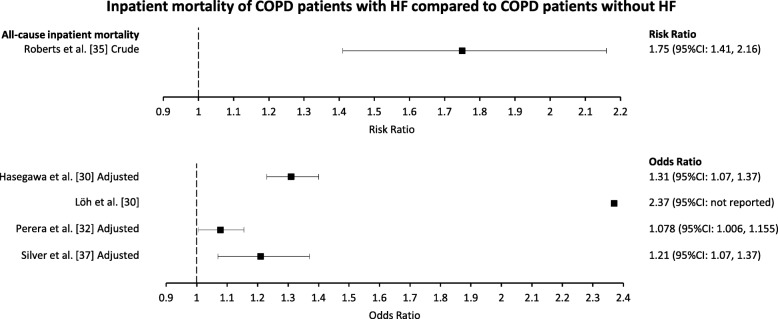
Estimates of the effect of HF comorbidity on the inpatient all-cause mortality of COPD patients. Heart failure (HF). Chronic obstructive pulmonary disease (COPD)

All studies reporting all-cause inpatient mortality were for deaths following COPD-related hospital admission and compared COPD patients with HF with those without HF (Fig. 9). Roberts et al. reported a crude risk ratio of 1.75 (95%CI: 1.41, 2.16) [43]. Löh et al. reported an OR of 2.37 (95%CI and adjustment status not reported) [50]. Hasegawa et al. reported an adjusted OR of 1.31 (95%CI: 1.23, 1.40) [37]. Perera et al. reported an adjusted OR of 1.08 (95%CI: 1.01, 1.16) for [40]. Silver et al. reported an adjusted OR of 1.21 (95%CI: 1.07, 1.37) [45]. Meta-analysis was not conducted as the majority of estimates were adjusted ORs, which are not compatible with meta-analysis methodology [24].

## Discussion

In this systematic review, the effect of HF comorbidity on hospitalisation and mortality within the COPD population was investigated. Hospitalisation, rehospitalisation, mortality, and inpatient mortality were all more common in COPD patients with HF than in COPD patients without HF. Risk of all-cause mortality was 1.61 times higher in COPD patients with HF than in COPD patients without HF, when COPD was diagnosed using spirometry.

### Hospitalisation and rehospitalisation

COPD patients with HF experienced greater hospitalisation, defined as A&E visits and/or admission to inpatient care, than COPD patients without HF. Schwab et al. reported significantly higher all-cause and COPD-related hospitalisation of COPD patients with HF compared with those without HF [49]. Santibáñez et al. reported significantly higher COPD-related hospitalisation of COPD patients with HF compared with those without HF [32]. The odds of COPD-related hospitalisation only slightly attenuated following adjustment. Both the crude and adjusted estimates came with large confidence intervals; however, both remained statistically significant [32]. Meanwhile, Boudestein et al. found that COPD patients with HF experienced around 40% more COPD- or cardiovascular-related hospitalisation compared with those without HF [13]. There was no attenuation following adjustment; however, both the crude and adjusted estimates came with large confidence intervals and were statistically insignificant [13]. It is likely that HF increases hospitalisation for COPD patients; however, there was considerable variance in many of the estimates that may be related to heterogeneity in the COPD populations studied.

Rehospitalisation, defined as repeat A&E visits and/or repeat admission to inpatient care, was more common in COPD patients with HF than in COPD patients without HF. Three estimates of 30-day COPD-related rehospitalisation did not reach statistical significance [36, 39, 41], while three more estimates showed statistically higher 30-day COPD-related rehospitalisation in COPD patients with HF compared with those without HF [39, 42]. Lau et al. found statistically higher 30-day rehospitalisation in their derivation cohort for both crude and adjusted estimates; however, the crude estimate for their validation cohort failed to reach statistical significance [39]. The 30-day COPD-related rehospitalisation estimate from Yeatts et al. failed to reach statistical significance; however, when follow-up was extended to 1-year, Yeatts et al. found statistically higher COPD-related rehospitalisation in COPD patients with HF compared with those without HF [36]. Chen et al. also found significantly higher 1-year COPD-related rehospitalisation in COPD patients with HF compared with those without HF [34]. Genao et al. followed patients for 3 years, finding increased all-cause and COPD-related rehospitalisation for COPD patients with HF compared with those without HF [35]. These estimates attenuated, but remained significant following adjustment [35].

The most evidence was available for COPD-related hospitalisation and rehospitalisation. COPD-related hospitalisation and rehospitalisation were higher in COPD patients with HF compared with those without HF, suggesting that HF increases severe AECOPD. This is supported by Criner et al. who reported the average number of exacerbations per person-year of follow-up was 1.5 times higher in COPD patients with HF compared with those without HF [9]. This is further supported by Cerezo Lajas et al. who reported that COPD patients with destabilised HF were at 5.25 times greater odds (95%CI: 1.11, 24.75) of frequent rehospitalisation, defined as ≥2 rehospitalisation within 30 days of index hospitalisation [8]. Many previous studies have investigated the effect of a compound variable representing ‘cardiovascular comorbidities’ on AECOPD incidence and severity; however, the heterogeneity of the various cardiovascular conditions included within such a compound variable may explain why these studies have found conflicting evidence [11, 53, 54].

The studies included in this review [32, 34–36, 39, 41, 42, 49], and others [8, 9], suggest that HF comorbidity increases COPD-related secondary care utilisation. The magnitude of this effect is more difficult to assess– as four different measures were used to evaluate hospitalisation or rehospitalisation (rate ratio, risk ratio, OR, HR), along with four different follow-up periods (30-day, 90-day, 1-year, 3-year) for rehospitalisation, with none having enough instances to enable meta-analysis. The effect of HF on COPD-related hospitalisation and rehospitalisation may be different in the short- vs long-term, in different COPD phenotypes, and/or with type and severity of HF, and future studies should investigate these possibilities.

### Mortality

The effect of HF comorbidity on all-cause mortality of COPD patients was the most reported outcome in this review. Roberts et al. reported significantly higher crude risk of all-cause mortality for COPD patients with HF compared with those without HF [43]. Kaszuba et al. and Santibáñez et al. both reported significantly higher odds of all-cause mortality in COPD patients with HF compared with those without HF, with the OR attenuating, but remaining significant, following adjustments [32, 38]. Twenty-four estimates, reported in 15 papers, of the effect of HF comorbidity on all-cause mortality of COPD patients were presented as HR. Only five of these estimates, all adjusted, failed to reach statistical significance [13, 44, 48, 51, 52]. Three different measures were used to evaluate mortality (risk ratio, OR, HR). Only HR had enough estimates to attempt a meta-analysis; however, when including all available data heterogeneity was too high to consider a pooled estimate. Only when limiting the analysis to studies that explicitly required spirometry for the diagnosis of COPD was heterogeneity low enough to consider a pooled estimate. In COPD patients diagnosed with spirometry, having HF resulted in a 1.61 times greater risk of all-cause mortality compared to COPD patients without HF; however, there was strong evidence of publication bias favouring studies showing an increased risk of mortality related to HF comorbidity.

Five studies reported estimates for all-cause inpatient mortality of COPD patients with HF compared with those without HF. All studies found significantly increased inpatient mortality for COPD patients with HF compared with those without HF [37, 40, 43, 45, 50]. Roberts et al. found that COPD patients with HF had 1.75 times the risk for all-cause inpatient mortality than those without HF [43]. In their conference abstract, Löh et al. did not report a 95%CI for their OR estimate of 2.37, nor did they explicitly state whether the estimate was crude or adjusted [50]. Excluding Löh et al., studies reporting OR estimated between 7 and 31% higher odds of all-cause mortality for COPD patients with HF compared with those without HF following adjustments [37, 40, 45]. Two measures were used to evaluate inpatient mortality (risk ratio, OR); however, three out of the four OR reported were explicitly adjusted and therefore not eligible for meta-analysis.

The COPD populations investigated were especially heterogeneous in terms of smoking history and severity of airflow limitation. For example, the cohort investigated in Ahn et al. ranged from no history of smoking to over 50 pack-years of smoking history [27], and the cohort investigated in Almagro et al. consisted of 21.6% patients with mild airflow limitation, 44.8% patients with moderate airflow limitation, 44% patients with severe airflow limitation, and 11% patients with very severe airflow limitation [28]. It may be that HF exerts greater effects on all-cause mortality in some COPD phenotypes than in others. Additionally, type and severity of HF may influence this effect. Future research should investigate these effects on the all-cause mortality of the COPD population.

### COPD comorbidity in HF patients

This work complements previous works that investigated the opposite relationship, namely the effect of comorbid COPD on hospitalisation and mortality of HF patients. Dunlay et al. and Wang et al. both found increased all-cause hospitalisation for HF patients with COPD comorbidity [55, 56]. Gulea et al. reported greater risk for 30-day all-cause, respiratory-related, and cardiovascular-related rehospitalisation following an index HF-related hospitalisation in HF patients with COPD comorbidity compared with those without COPD [57]. Similarly, Rushton et al. conducted a meta-analysis finding that COPD comorbidity significantly increased all-cause mortality in HF patients (pooled HR: 1.39, 95%CI: 1.2–1.6; I^2^ = 37.7%) [58]. These findings are in line with ours in demonstrating increased morbidity and mortality due to comorbidity in both the COPD and HF populations.

### Strengths and limitations

In the included studies, there was a low risk of bias in areas where risk of bias could be determined; however, there were many areas in which reporting was incomplete leading to unknown risk of bias. There was evidence of publication bias favouring studies showing an effect in the one meta-analysis that was conducted.

There was considerable heterogeneity in the hazard ratio estimates of all-cause mortality, preventing meta-analysis using all available data. Heterogeneity appeared to result from the use of electronic diagnostic coding for the identification of COPD patients, where validity of coding is uncertain. Diagnostic coding can be validated in electronic record systems; however, the process is labour-intensive and requires sending and receiving back questionnaires from the coding clinicians [6, 7, 59]. Additionally, many studies relied solely upon diagnostic coding for the identification of HF, meaning the validity of the HF diagnosis is unknown. COPD and HF share many symptoms and misdiagnosis may result. HF is often underdiagnosed in patients with COPD [3], which may impact the implications of these results, as some patients classified as COPD without HF may have in fact had HF.

Meta-analysis was not possible for the outcomes hospitalisation, rehospitalisation, and inpatient mortality due to insufficient data. A number of studies were found during searching that reported only adjusted OR for the outcomes of interest in this review, which are not compatible with meta-analysis [24].

## Conclusions

Evidence suggests that HF comorbidity increases COPD-related secondary care utilisation and all-cause mortality in the COPD population; however, meta-analysis was only appropriate for the effect of HF on all-cause mortality of COPD patients, diagnosed using spirometry. Risk of all-cause mortality was 1.61 times higher for COPD patients with HF compared to those without HF, when COPD was diagnosed using spirometry. Delayed diagnosis of HF in COPD patients and under-treatment of cardiovascular conditions in the COPD population may contribute to this increased morbidity and mortality. HF comorbidity may have a greater effect on morbidity and mortality in some COPD phenotypes than in others, or may differ based on type and severity of HF, and future work should investigate this possibility. Creation of bespoke guidelines, pathways, and clinical and patient education for the diagnosis and management of HF in the presence of COPD, and vice versa, are needed to improve patient outcomes.

## Supplementary information

**Additional file 1: Figure S1.** Search of MEDLINE on 05 February 2019. **Figure S2.** Search of Embase on 05 February 2019. **Table S1.** Overview of included literature.

## Data Availability

This was a systematic review of previously published literature.

## Abbreviations

CI: Confidence interval
COPD: Chronic obstructive pulmonary disease
HF: Heart failure
HR: Hazard ratio
IQR: Interquartile range
OR: Odds ratio
SD: Standard deviation
