# Supplementary material for: Hospitalisation and mortality in patients with comorbid COPD and heart failure: a systematic review and meta-analysis
Source: Respir Res. 2020 Feb 14;21:54. doi: 10.1186/s12931-020-1312-7 (PMC7023777; doi:10.1186/s12931-020-1312-7)
Supplement: Supplementary file 1 — Additional file 1: Figure S1. Search of MEDLINE on 05 February 2019. Figure S2. Search of Embase on 05 February 2019. Table S1. Overview of included literature. [file 12931_2020_1312_MOESM1_ESM.docx]

# Supplementary Material

[Supplementary Figure 1. Search of MEDLINE on 05 February 2019. 2](#_Toc8726899)

[Supplementary Figure 2. Search of Embase on 05 February 2019. 3](#_Toc8726900)

[Supplementary Table 1. Overview of included literature. 4](#_Toc8726906)


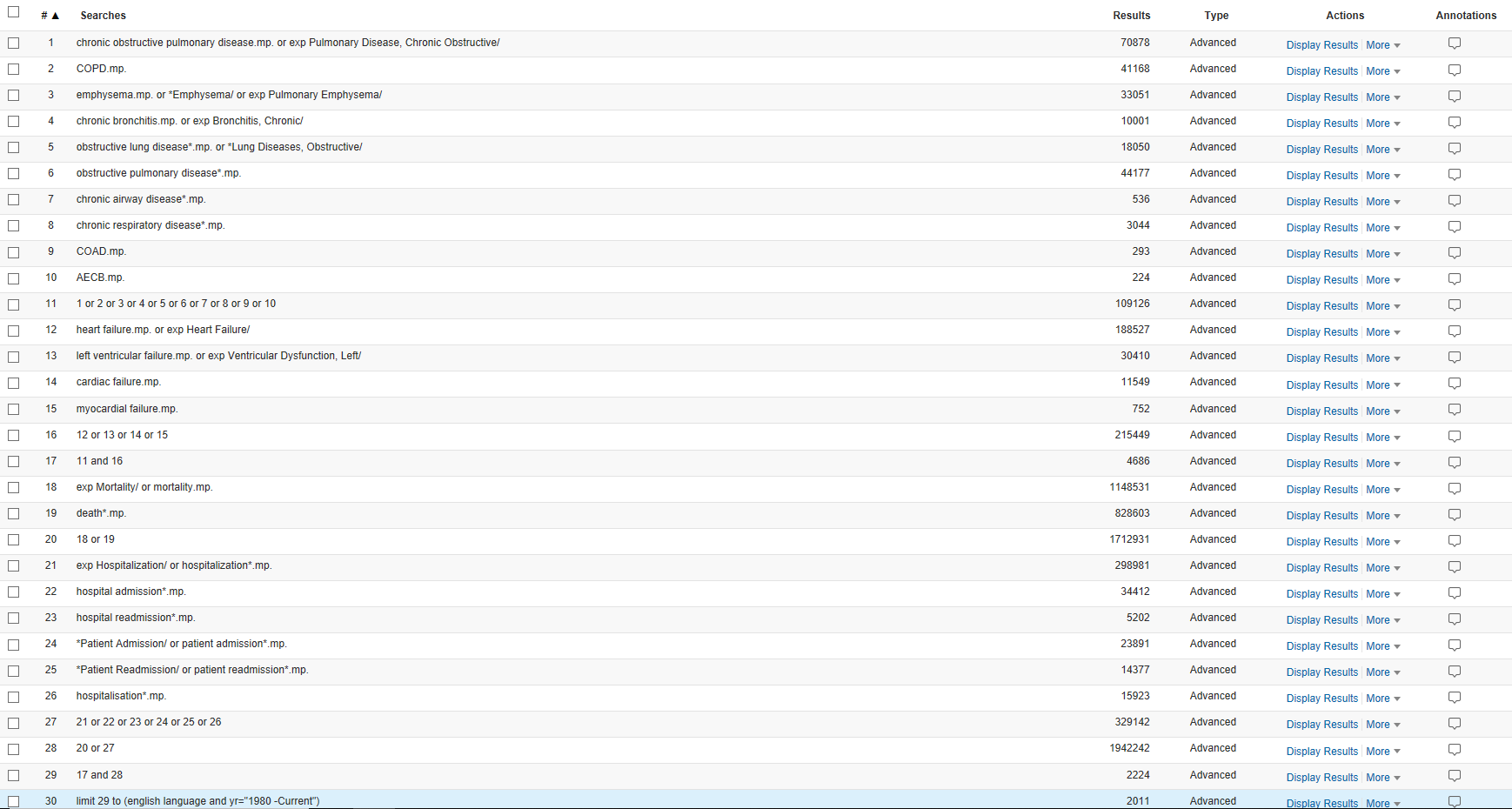


Supplementary Figure 1. Search of MEDLINE on 05 February 2019.


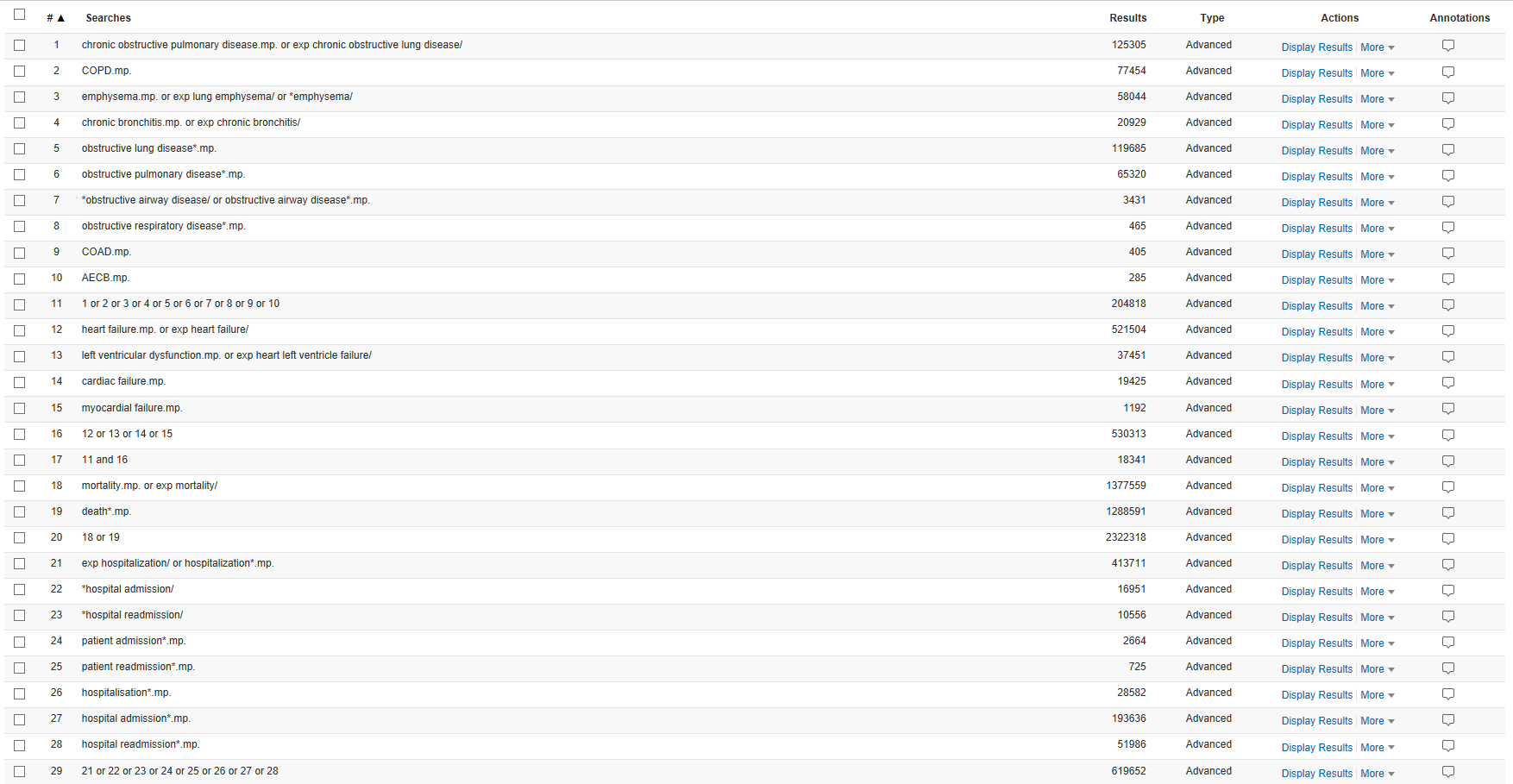

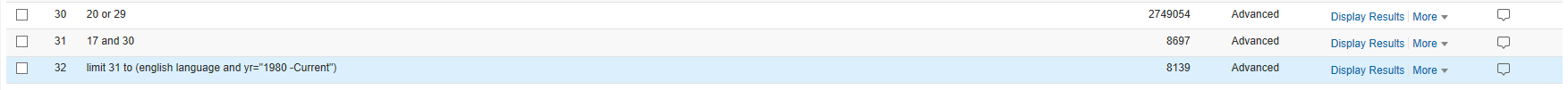


Supplementary Figure 2. Search of Embase on 05 February 2019.

Supplementary Table 1. Overview of included literature.

| **Study** | **Design** | **Inclusion Criteria** | **Exclusion Criteria** | **Patients** | **Length of Follow-Up** | **Outcome(s)*** | **Setting** |
| --- | --- | --- | --- | --- | --- | --- | --- |
| Abukhalaf et al. 2018 [1] | Retrospective Cohort | Pre- or post-bronchodilator FEV_1_/FVC < 0.70; ≥40 years; >20 pack-years smoking; clinical diagnosis of COPD | Diagnosis of bronchiolitis, granulomatosis with polyangitis, or asthma  Post‐bronchodilator FEV_1_/FVC > 0.70 | 512 | Median (IQR):  3 years (1.4-5.2) | Hazard ratio for mortality adjusted for lung function, BMI, smoking, comorbidities, exacerbation history | University of New Mexico  Health Sciences Center (USA) |
| Ahn et al. 2015 [2] | Retrospective Cohort | Post-bronchodilator  FEV_1_/FVC < 0.70  Left heart failure was defined as ≤40% ejection fraction | Abnormal chest x-ray; no spirometry | 229 | Average (SD):  6.48 years (4.09) | Univariate hazard ratio for mortality | Ajou University Hospital (Republic of Korea) |
| Almagro et al. 2012 [3]  EPOC en Servicios de Medicina Interna (ESMI) study | Longitudinal cohort | Post-bronchodilator FEV_1_/FVC < 0.70; post-bronchodilator FEV_1_ < 80% predicted; ≥40 years; admission for COPD exacerbation | Asthma; bronchiectasis; pulmonary oedema; pneumonia; no spirometry; admission for reason other than COPD exacerbation | 606 | 0.23 years (12 weeks) | Univariate hazard ratio for mortality | 70 A&E and internal medicine services  (Spain) |
| Belloli et al. 2011 [4]  Abstract | Retrospective Cohort | COPD coded in registry | ND | 1132 | Median (IQR):  1.55 years (0.74-2.5) | Hazard ratio for mortality adjusted for age, sex, lung function, comorbidities, NT-proBNP | University of Pittsburgh COPD Patient Registry and Molecular and Cellular Determinants of Disease Heterogeneity in COPD Registry  (USA) |
| Bertens et al. 2010 [5]  Abstract | Retrospective Cohort | COPD coded in general practice medical records; aged 45 | ND | 2230 | ND | Hazard ratio for mortality adjusted for age, sex, comorbidities, medications | 23 general practices (Netherlands) |

*Supplementary Table 1. Overview of included literature. Continued.*

| **Study** | **Design** | **Inclusion Criteria** | **Exclusion Criteria** | **Patients** | **Length of Follow-Up** | **Outcome(s)*** | **Setting** |
| --- | --- | --- | --- | --- | --- | --- | --- |
| Boudestein et al. 2009 [6] | Prospective Cohort | Clinical diagnosis of COPD by general practitioner; ≥65 years old; post-bronchodilator FEV_1_/FVC < 0.70  HF identified by expert panel including two cardiologist using European Society of Cardiology (ESC) guidelines classification as systolic, diastolic, or right-sided; systolic ≤45% left ventricular ejection fraction | Previous cardiologist-confirmed diagnosis of HF | 404 | Average (SD):  4.2 years (1.4) | Hazard ratio for mortality adjusted for age, sex, smoking, comorbidities, medications | Recruited from 51 general practices; study took place at University Medical Center Utrecht  (Netherlands) |
| Carter et al. 2019 [7] | Prospective Cohort | Age ≥18 years old; COPD admission coded using the International Classification of Disease 10th edition (ICD-10) and Office of Population Censuses and Surveys Classification of Interventions and Procedures (OPCS-4) | ND | 31,646 | Average (SD):  5.2 years (3.6) | Hazard ratio for mortality adjusted for age, sex, comorbidities, ethnicity | 7 NHS hospitals in North West of England  (UK) |
| Chen et al. 2009 [8] | Retrospective Cohort | ≥40 years old; hospitalised for COPD where COPD was coded using International Classification of Diseases, Version 9 (ICD-9) as one of the first five diagnoses for the admission | Death prior to discharge from initial hospitalisation | 108,726 | Maximum of 1 year | Hazard ratio for COPD readmission adjusted for sex, age, Canadian province, length of stay at baseline | Health  Person-Oriented Information (HPOI) Database  (Canada) |

*Supplementary Table 1. Overview of included literature. Continued.*

| **Study** | **Design** | **Inclusion Criteria** | **Exclusion Criteria** | **Patients** | **Length of Follow-Up** | **Outcome(s)*** | **Setting** |
| --- | --- | --- | --- | --- | --- | --- | --- |
| Divo et al. 2012 [9]  BODE cohort [10] | Prospective Cohort | Post-bronchodilator FEV_1_/FVC < 0.70; history of smoking >10 or >20 pack-years, there is a discrepancy between the number reported in Divo et al. and the number reported in the description of the BODE cohort [10] cited by Divo et al. | Asthma; no spirometry; no six-minute walk test; myocardial infraction in previous 4 months; unstable angina; congestive HF (New York Heart Association class III or IV) | 1,659 | Median (IQR):  4.25 years (2.3-6.5) | Hazard ratio for mortality adjusted for age, sex, race, BMI, lung function, dyspnoea, exercise capacity | Pulmonary clinics  (USA and Spain) |
| Genao et al. 2015 [11] | Retrospective Cohort | ≥ 65 years old; AECOPD admission coded in the primary position of inpatient claim or emergency visit using International Classification of Diseases, Ninth Revision, Clinical Modification (ICD-9-CM); continuously enrolled in Medicare fee-for-service for at least 12 months prior to and after (barring death) initial hospitalisation | Death prior to discharge from initial hospitalisation | 52,741 | Maximum of 3 years | Hazard ratios for mortality, for all-cause readmission, and for COPD-related readmission adjusted for age, sex, comorbidities, Medicare and Medicaid eligibility, malnutrition, race | Medicare fee-for-service claims data from U.S. Centers for Medicare and Medicaid Services  (USA) |

*Supplementary Table 1. Overview of included literature. Continued.*

| **Study** | **Design** | **Inclusion Criteria** | **Exclusion Criteria** | **Patients** | **Length of Follow-Up** | **Outcome(s)*** | **Setting** |
| --- | --- | --- | --- | --- | --- | --- | --- |
| Hasegawa et al. 2014 [12] | Retrospective Cohort | > 40 years old; COPD in any diagnostic position following emergency hospital admission | ND | 172,707 | ND | Odds ratio for all-cause inpatient mortality | Diagnosis Procedure Combination (DPC) database  (Japan) |
| Hoiseth et al. 2016 [13] | Prospective Cohort | Admission for dyspnoea as primary complaint; adjudication of AECOPD based on GOLD guidelines; Adjudication of HF according to ESC guidelines | Disseminated malignant disease; inability to cooperate | 75 | Median (IQR):  1.27 years (0.83-1.9) | Hazard ratio for long-term mortality adjusted for age, BMI, systolic blood pressure, pulmonary attenuation, pH, troponin levels, insulin use, aldosterone antagonist use | Akershus Cardiac  Examination (ACE) 2 Study  (Norway) |
| Kaszuba et al. 2018 [14] | Prospective Cohort | ≥ 35 years old; COPD coded using ICD-10 codes in at least one primary or secondary care consultation  HF coded as ICD-10 code I50 | Left Blekinge County during observation period | 984 | 7 years | Odds ratio for all-cause mortality | Blekinge County council health care register  (Sweden) |
| Kim et al. 2009 [15] | Retrospective Cohort | COPD admission coded in the first position of inpatient or emergency room visit using ICD-9 codes | ND | 482 | Median (IQR):  3.09 years (1.2-4.3) | Hazard ratio for mortality adjusted for age, sex, marital status, comorbidities, exacerbation history | Research Patient Data  Repository at Partners HealthCare  (USA) |

*Supplementary Table 1. Overview of included literature. Continued.*

| **Study** | **Design** | **Inclusion Criteria** | **Exclusion Criteria** | **Patients** | **Length of Follow-Up** | **Outcome(s)*** | **Setting** |
| --- | --- | --- | --- | --- | --- | --- | --- |
| Lainscak et al. 2009 [16]  Abstract | Retrospective Cohort | COPD and HF coded in registry by ICD-10; diagnosis ascertained by review of medical records | ND | 960 | 2.8 years | Hazard ratio for mortality adjusted (variables for adjustment not reported) | Central Population Registry  (Slovenia) |
| Lau et al. 2017 [17] | Retrospective Cohorts  Derivation and validation cohorts | > 40 years old; index admission for COPD coded by ICD-10 | ND | 339,389  +  258,113 | 30 days | Odds ratio for 30-day COPD-related readmission; Odds ratio for inpatient all-cause mortality | State Inpatient Database  (USA) |
| Löh et al. 2014 [18]  Abstract | Retrospective Cohort | Primary diagnosis of COPD using ICD-10 codes; Primary diagnosis of respiratory failure with secondary diagnosis of COPD using ICD-10 codes | ND | 995,044 | ND | Odds ratio for inpatient all-cause mortality | German-diagnosis-related-groups (G-DRG) database from German Federal Statistical Office  (Germany) |
| Maters et al. 2014 [19] | Prospective Cohort | COPD diagnosed according to GOLD guidelines; stable COPD for at least 6 weeks; available medical history | Inability to fill out questionnaires, perform cycle ergometry, and/or perform spirometry | 224 | 4.2 years | Univariate hazard ratio for mortality | Center for Rehabilitation of the University  Medical Center Groningen (UMCG)  (Netherlands) |
| Miller et al. 2013 [20]  ECLIPSE study [21]  NCT00292552 | Prospective Cohort | COPD patients aged 40-75 years; post-bronchodilator FEV_1_/FVC < 0.70; post-bronchodilator FEV_1_ < 80% predicted; smoking history ≥10 pack-years | Ability to comply with protocol; availability for study visits over three years; presence of a respiratory disorder other than COPD; active rheumatoid arthritis or inflammatory bowel disease; exacerbation within 4 weeks of enrolment | 2,164 | 3 years | Univariate hazard ratio for mortality | Multinational |

*Supplementary Table 1. Overview of included literature. Continued.*

| **Study** | **Design** | **Inclusion Criteria** | **Exclusion Criteria** | **Patients** | **Length of Follow-Up** | **Outcome(s)*** | **Setting** |
| --- | --- | --- | --- | --- | --- | --- | --- |
| Perera et al. 2012 [22] | Retrospective Cohort | ≥ 40 years old; ICD-9 code for COPD with concurrent diagnosis for pneumonia or mechanical ventilation | ND | 1,254,703 | ND | Odds ratio for all-cause inpatient mortality | Agency for Healthcare Research and Quality (AHRQ) Healthcare Cost and Utilization Project (HCUP) Nationwide Inpatient Sample for 2006  (USA) |
| Roberts et al. 2011 [23]  UK National COPD Audit 2008 [24] | Prospective Cohort | COPD admission identified through the audit | ND | 9169 | 90 days | Unadjusted risk ratio for all-cause 90 day mortality; unadjusted risk ratio for all-cause 90 readmission | UK National COPD Audit 2008 |
| Santibáñez et al. 2016 [25] | Retrospective Cohort | ≥ 35 years old; COPD coded using International Classification of Primary Care; post-bronchodilator FEV_1_/FVC < 0.70 | post-bronchodilator FEV_1_/FVC > 0.70 or not recorded | 900 | 1 year | Odds ratio for COPD-related hospitalisation; Odds ratio for inpatient mortality | Electronic clinical databases in province of Cantabria  (Spain) |
| Schwab et al. 2017 [26] | Retrospective Cohort | ≥2 medical claims with a COPD diagnosis recorded using ICD-9 in primary or secondary position; 40-89 years old at diagnosis; continuously enrolled in Medicare Advantage plans with Prescription Drug benefits for 12 months prior to index date and 24 months after index date | Enrolment in Administrative Services Only or commercial plan with data sharing restrictions; ≥1 medical claim with cystic fibrosis, pulmonary tuberculosis, or malignant neoplasms coded using ICD-9 at any position during study period | 52,643 | 2 years | Rate ratios for all-cause and COPD-related hospitalisations adjusted for age, sex, geographic location, influenza vaccination status, comorbidities, exacerbation history, pre-index all-cause hospitalisation/ emergency visits/ outpatient visits | US national health plan (Humana Inc., Louisville, KY, USA) |

*Supplementary Table 1. Overview of included literature. Continued.*

| **Study** | **Design** | **Inclusion Criteria** | **Exclusion Criteria** | **Patients** | **Length of Follow-Up** | **Outcome(s)*** | **Setting** |
| --- | --- | --- | --- | --- | --- | --- | --- |
| Sharif et al. 2014 [27] | Retrospective Cohort | 40-64 years old; hospitalised with primary discharge diagnosis of COPD using ICD-9 codes | Incomplete data for 12 months prior to index hospitalisation; transferred to long-term facility; nonspecific bronchitis and asthma discharge codes | 8,236 | 30 days | Odds ratio for 30-day all-cause readmission | The Clinformatics Data Mart, managed by OPTUMInsight of Minneapolis, MN  (USA) |
| Silver et al. 2010 [28] | Cross-sectional | Hospitalised for COPD exacerbation in primary diagnostic position | NS | 69,841 | ND | Odds ratio for all-cause inpatient mortality | Premier’s Perspective Comparative Database  (USA) |
| Simmering et al. 2016 [29] | Retrospective cohort | ≥ 40 years old; COPD in first diagnostic position for index hospitalisation; discharged alive; complete covariate information | Inpatient death at index hospitalisation | 286,313 | 30 days | Odds ratio for all-cause 30-day rehospitalisation | Healthcare Cost and Utilization Project (HCUP) State Inpatient Database for California  (USA) |
| Slenter et al. 2013 [30] | Retrospective Cohort | COPD admission coded using ICD-9; FEV_1_/FVC < 0.70 and FEV_1_ reversibility <11% *or* clinical diagnosis; treatment with systemic glucocorticoids during admission | Patients transferred into hospital following initial admission at another hospital | 260 | 1 year | Hazard ratio of 1 year mortality adjusted for age, sex, prior COPD-related hospitalisation in last two years, PaCO_2_ at admission, urea level at admission | Pulmonary wards of Maastricht University Hospital  (Netherlands) |
| Yeatts et al. 2013 [31] | Retrospective Cohort | ≥45 years old; ≥1 COPD-related emergency visit coded in the first or second discharge position using ICD-9 | Bronchitis | 33,799 | 30 days or 1 year | Unadjusted risk ratios for COPD-related readmission within 30 days or 1 year | North Carolina Public Health Data Group and the North Carolina Disease Event Tracking and Epidemiologic Collection Tool (NC DETECT) surveillance system  (USA) |

*Details which outcome(s) were used in these analyses and therefore may not reflect the full scope of the individual study. *Abbreviations:* Accident and emergency (A&E). Body mass index (BMI). Chronic obstructive pulmonary disease (COPD). Forced expiratory volume in one second (FEV_1_). Forced vital capacity (FVC). Interquartile range (IQR). National Health Service (NHS). Not described (ND). N-terminal pro brain natriuretic peptide (NT-proBNP). Partial pressure of carbon dioxide in the arterial blood (PaCO_2_). Standard deviation (SD). United Kingdom (UK). United States of America (USA).

**References**

1. Abukhalaf J, Davidson R, Villalobos N, Meek P, Petersen H, Sood A, Tesfaigzi Y, Vazquez Guillamet R. Chronic obstructive pulmonary disease mortality, a competing risk analysis. *Clinical Respiratory Journal* 2018: 12(11): 2598-2605.

2. Ahn YH, Lee KS, Park JH, Jung JH, Lee M, Jung YJ, Chung WY, Sheen S, Park KJ, Kim DJ, Kang DR, Lee JD, Yoon S, Jin XJ, Yang HM, Lim HS, Park JS, Shin JH, Tahk SJ. Independent risk factors for mortality in patients with chronic obstructive pulmonary disease who undergo comprehensive cardiac evaluations. *Respiration* 2015: 90(3): 199-205.

3. Almagro P, Cabrera FJ, Diez J, Boixeda R, Alonso Ortiz MB, Murio C, Soriano JB. Comorbidities and short-term prognosis in patients hospitalized for acute exacerbation of COPD: The EPOC en servicios de medicina interna (ESMI) study. *Chest* 2012: 142(5): 1126-1133.

4. Belloli EA, Stamm JA, Zhang Y, Gladwin MT, Sciurba FC. N-terminal pro brain natriuretic peptide in a large chronic obstructive pulmonary disease cohort: Clinical characterization and impact on survival. *American Journal of Respiratory and Critical Care Medicine* 2011: 183(1 MeetingAbstracts).

5. Bertens LCM, Van Mourik Y, Guder G, Hoes AW, Rutten FH. Gender modifies the effect of heart failure on survival in patients with COPD. *European Journal of Heart Failure, Supplement* 2010: 9(SUPPL. 1): S105.

6. Boudestein LCM, Rutten FH, Cramer MJ, Lammers JWJ, Hoes AW. The impact of concurrent heart failure on prognosis in patients with chronic obstructive pulmonary disease. *European Journal of Heart Failure* 2009: 11(12): 1182-1188.

7. Carter P, Lagan J, Fortune C, Bhatt DL, Vestbo J, Niven R, Chaudhuri N, Schelbert EB, Potluri R, Miller CA. Association of Cardiovascular Disease With Respiratory Disease. *J Am Coll Cardiol* 2019.

8. Chen Y, Li Q, Johansen H. Age and sex variations in hospital readmissions for COPD associated with overall and cardiac comorbidity. *International Journal of Tuberculosis and Lung Disease* 2009: 13(3): 394-399.

9. Divo M, Cote C, Pinto-Plata VM, De Torres J, Casanova C, Marin J, Zulueta J, Zagaceta J, Cabrera Lopez C, Celli BR. Comorbidities, gender and mortality differences in patients with COPD. *American Journal of Respiratory and Critical Care Medicine* 2012: 185(MeetingAbstracts).

10. Celli B, Cote C, Marin J, Casanova C, Montes de Oca M, Mendez M, Pinto Plata V, Cabral H. The Body-Mass Index, Airflow Obstruction, Dyspnea, and Exercise Capacity Index in Chronic Obstructive Pulmonary Disease. *N Engl J Med* 2004: 350: 1005-1012.

11. Genao L, Durheim MT, Mi X, Todd JL, Whitson HE, Curtis LH. Early and long-term outcomes of older adults after acute care encounters for chronic obstructive pulmonary disease exacerbation. *Annals of the American Thoracic Society* 2015: 12(12): 1805-1812.

12. Hasegawa W, Yamauchi Y, Yasunaga H, Sunohara M, Jo T, Matsui H, Fushimi K, Takami K, Nagase T. Factors affecting mortality following emergency admission for chronic obstructive pulmonary disease. *BMC Pulmonary Medicine* 2014: 14(1): 151.

13. Hoiseth AD, Brynildsen J, Hagve TA, Christensen G, Soyseth V, Torbjorn O, Rosjo H. The influence of heart failure co-morbidity on high-sensitivity troponin T levels in COPD exacerbation in a prospective cohort study: Data from the Akershus cardiac examination (ACE) 2 study. *Biomarkers* 2016: 21(2): 173-179.

14. Kaszuba E, Odeberg H, Rastam L, Halling A. Impact of heart failure and other comorbidities on mortality in patients with chronic obstructive pulmonary disease: a register-based, prospective cohort study. *BMC family practice* 2018: 19(1): 178.

15. Kim S, Clark S, Camargo Jr CA. Mortality after an emergency department visit for exacerbation of chronic obstructive pulmonary disease. *COPD: Journal of Chronic Obstructive Pulmonary Disease* 2006: 3(2): 75-81.

16. Lainscak M, Von Haehling S, Doehner W, Sarc I, Jeric T, Ziherl K, Kosnik M, Suskovic S, Anker SD. Chronic heart failure in patients with acute exacerbation of chronic obstructive pulmonary disease: Prevalence, clinical characteristics, treatment and mortality. *Journal of Cardiac Failure* 2009: 15(6 SUPPL. 1): S99.

17. Lau CSM, Siracuse BL, Chamberlain RS. Readmission after COPD exacerbation scale: Determining 30-day readmission risk for COPD patients. *International Journal of COPD* 2017: 12: 1891-1902.

18. Loh B, Von Der Beck D, Korfei M, Seeger W, Gunther A. Cormorbidities and ventilator therapy impact on mortality of COPD patients in German hospitals-an analysis of ICD statistics. *European Respiratory Journal* 2014: 44(SUPPL. 58).

19. Maters GA, De Voogd JN, Sanderman R, Wempe JB. Predictors of all-cause mortality in patients with stable copd: Medical co-morbid conditions or high depressive symptoms. *COPD: Journal of Chronic Obstructive Pulmonary Disease* 2014: 11(4): 468-474.

20. Miller J, Edwards LD, Agusti A, Bakke P, Calverley PMA, Celli B, Coxson HO, Crim C, Lomas DA, Miller BE, Rennard S, Silverman EK, Tal-Singer R, Vestbo J, Wouters E, Yates JC, Macnee W. Comorbidity, systemic inflammation and outcomes in the ECLIPSE cohort. *Respiratory Medicine* 2013: 107(9): 1376-1384.

21. Vestbo J, Anderson W, Coxson HO, Crim C, Dawber F, Edwards L, Hagan G, Knobil K, Lomas DA, MacNee W, Silverman EK, Tal-Singer R, investigators E. Evaluation of COPD Longitudinally to Identify Predictive Surrogate End-points (ECLIPSE). *Eur Respir J* 2008: 31(4): 869-873.

22. Perera PN, Armstrong EP, Sherrill DL, Skrepnek GH. Acute exacerbations of COPD in the United States: Inpatient burden and predictors of costs and mortality. *COPD: Journal of Chronic Obstructive Pulmonary Disease* 2012: 9(2): 131-141.

23. Roberts CM, Stone RA, Lowe D, Pursey NA, Buckingham RJ. Co-morbidities and 90-day outcomes in hospitalized COPD exacerbations. *COPD: Journal of Chronic Obstructive Pulmonary Disease* 2011: 8(5): 354-361.

24. National COPD Audit 2008. 2008 [cited 2019 15 May]; Available from: <https://www.rcplondon.ac.uk/projects/outputs/national-copd-audit-2008>

25. Santibanez M, Garrastazu R, Ruiz-Nunez M, Helguera JM, Arenal S, Bonnardeux C, Leon C, Garcia-Rivero JL. Predictors of hospitalized exacerbations and mortality in chronic obstructive pulmonary disease. *PLoS ONE* 2016: 11(6): e0158727.

26. Schwab P, Dhamane AD, Hopson SD, Moretz C, Annavarapu S, Burslem K, Renda A, Kaila S. Impact of comorbid conditions in COPD patients on health care resource utilization and costs in a predominantly medicare population. *International Journal of COPD* 2017: 12: 735-744.

27. Sharif R, Parekh TM, Pierson KS, Kuo YF, Sharma G. Predictors of early readmission among patients 40 to 64 years of age hospitalized for chronic obstructive pulmonary disease. *Annals of the American Thoracic Society* 2014: 11(5): 685-694.

28. Silver H, Blanchette CM, Roberts M, Petersen H, St Charles ME. Prevalence of comorbidities in patients hospitalized for COPD exacerbations and impact on impatient mortality and hospital expenditures. *American Journal of Respiratory and Critical Care Medicine* 2010: 181(1 MeetingAbstracts).

29. Simmering JE, Polgreen LA, Comellas AP, Cavanaugh JE, Polgreen PM. Identifying Patients With COPD at High Risk of Readmission. *Chronic obstructive pulmonary diseases (Miami, Fla)* 2016: 3(4): 729-738.

30. Slenter RHJ, Sprooten RTM, Kotz D, Wesseling G, Wouters EFM, Rohde GGU. Predictors of 1-year mortality at hospital admission for acute exacerbations of chronic obstructive pulmonary disease. *Respiration* 2013: 85(1): 15-26.

31. Yeatts KB, Lippmann SJ, Waller AE, Lich KH, Travers D, Weinberger M, Donohue JF. Population-based burden of COPD-related visits in the ED: Return ED visits, hospital admissions, and comorbidity risks. *Chest* 2013: 144(3): 784-793.
